# Crossroad between the Heat Shock Protein and Inflammation Pathway in Acquiring Drug Resistance: A Possible Target for Future Cancer Therapeutics

**DOI:** 10.3390/biomedicines11102639

**Published:** 2023-09-26

**Authors:** Prathap Somu, Nagaraj Basavegowda, Levin Anbu Gomez, Hulikunte Veeranna Jayaprakash, Gangadahosahalli Krishnegowda Puneetha, Akhilesh Kumar Yadav, Subhankar Paul, Kwang-Hyun Baek

**Affiliations:** 1 Department of Biotechnology and Chemical Engineering, School of Civil & Chemical Engineering, Manipal University Jaipur, Dehmi Kalan, Jaipur 303007, India; prathaps1987@gmail.com; 2 Department of Biotechnology, Yeungnam University, Gyeongsan 38451, Republic of Korea; nagarajb2005@yahoo.co.in; 3 Department of Biotechnology, School of Agriculture and Bioscience, Karunya Institute of Technology and Sciences (Deemed-to-be University), Karunya Nagar, Coimbatore 641114, India; levin@karunya.edu; 4 Department of Chemistry, Sri Siddhartha Institute of Technology, Maralur, Tumkur 572105, India; jayaprakashhv@ssit.edu.in; 5 Department of Botany, Yuvaraja’s College, University of Mysore, Mysuru 570005, India; puneethagk@gmail.com; 6 Department of Environmental Engineering and Management, Chaoyang University of Technology, Taichung 413310, Taiwan; yadavbasti@gmail.com; 7 Structural Biology and Nanomedicine Laboratory, Department of Biotechnology and Medical Engineering, National Institute of Technology, Rourkela 769008, India

**Keywords:** cancer, multidrug resistance, tumor microenvironment, interleukin, heat shock proteins, immunomodulatory effect

## Abstract

The development of multidrug resistance (MDR) against chemotherapeutic agents has become a major impediment in cancer therapy. Understanding the underlying mechanism behind MDR can guide future treatment for cancer with better therapeutic outcomes. Recent studies evidenced that crossroads interaction between the heat shock proteins (HSP) and inflammatory responses under the tumor microenvironment plays a pivotal role in modulating drug responsiveness and drug resistance through a complex cytological process. This review aims to investigate the interrelationship between inflammation and HSP in acquiring multiple drug resistance and investigate strategies to overcome the drug resistance to improve the efficacy of cancer treatment. HSP plays a dual regulatory effect as an immunosuppressive and immunostimulatory agent, involving the simultaneous blockade of multiple signaling pathways in acquiring MDR. For example, HSP27 shows biological effects on monocytes by causing IL10 and TNFα secretion and blocking monocyte differentiation to normal dendritic cells and tumor-associated macrophages to promote cancer progression and chemoresistance. Thus, the HSP function and immune-checkpoint release modalities provide a therapeutic target for a therapeutically beneficial approach for enhancing anti-tumor immune responses. The interconnection between inflammation and HSP, along with the tumor microenvironment in acquiring drug resistance, has become crucial for rationalizing the effect of HSP immunomodulatory activity with immune checkpoint blockade. This relationship can overcome drug resistance and assist in the development of novel combinatorial cancer immunotherapy in fighting cancer with decreasing mortality rates.

## 1. Introduction

Cancer includes a group of diseases that occur due to the uncontrolled division of the cells that can spread and invade throughout body parts. Cancer is the second most significant factor in morbidity worldwide, behind cardiovascular disease, and kills approximately 10 million people each year, according to the WHO (World Health Organization). Further, it is projected that 1,958,310 new cancer cases and 609,820 fatalities due to cancer emerge worldwide in 2023. The most common types of cancer are breast, lung, colorectal, and prostate cancers [1]. It has further been estimated that around 2030, the number of new cancer cases will rise by nearly 70% [2]. 

Currently practiced therapeutic intervention for cancer treatment includes radiation therapy, immunotherapy, surgery, chemotherapy, and targeted therapy. Radiation therapy and surgery have frequently been practiced to treat localized and non-metastatic cancers [3,4]. Chemotherapy is the most clinically practiced therapeutic intervention for treating cancer cells that have spread into distant organs in the whole body, which are no longer treated with localized therapeutic methods such as surgery and radiotherapy clinically [5,6]. The cytotoxic potential of these chemotherapeutic agents is due to their ability to interfere with DNA synthesis and mitosis of rapidly proliferating cells, thereby causing their death (rapid proliferation is one of the essential characteristics of neoplastic cells such as cancer cells). For instance, Taxol is a commonly used chemotherapy agent that hinders microtubule depolarization and hyper-stabilizes the microtubule. Another widely used and the most important chemotherapy agent is doxorubicin, which inhibits topoisomerase II, an essential enzyme for relaxing supercoils in DNA during cell division.

Despite the achievements made in cancer treatment, chemoresistance, or drug resistance to chemotherapeutic agents, has become a significant problem in cancer therapies and is responsible for most tumor relapses and poor prognosis [7]. The cytotoxic potential of these chemotherapeutic agents is due to their ability to interfere with the synthesis of DNA of rapidly dividing cells, which is non-specific in nature and thereby causes side-effects of the rapidly dividing healthy cells [8,9]. Recently, efforts have been made to develop more specific and targeted drugs that precisely target/block cancer growth and proliferation. Notable results have been obtained during the initial treatment for these chemotherapeutic agents; however, as the treatment proceeded, most patients developed resistance. For instance, there are reports on the recurrence and development of cancer in some patients after surgery and adjuvant chemotherapy S-1, an oral fluoropyrimidine [10]. Thus, the underlying mechanism for acquiring drug resistance must be understood to improve or develop new interventions and therapeutic strategies for better clinical outcomes. This book chapter describes the specific mechanisms involved in acquiring intrinsic and acquired drug resistance and contemporary techniques for fighting against drug resistance and improving anti-cancer efficacy.

## 2. Drug Resistance or Chemoresistance

Chemoresistance initiates from factors such as the genetic plasticity of cancer cells causing somatic mutations of drug targets, modified drug transport, altered drug metabolism, or dysregulated apoptosis. Further, cellular machinery like molecular chaperones (heat shock proteins), overexpression of anti-apoptotic proteins, detoxifying enzymes, and drug efflux transporters also help to acquire drug resistance [11,12,13]. Drug resistance may be an inherent and innate feature of the cancer cells or developed during treatment as a result of the adaption of tumors to counter drug exposure (acquired resistance). In both conditions, i.e., innate or acquired, resistance typically leads to treatment failure and tumor recurrence. 

In the cancer cells exposed to hypoxia, the surviving cells induce a multifaceted transcriptional response after the initial treatment to protect themselves from any such environment in the future [11,14]. HSPs are cellular machinery that primarily function as molecular chaperones that are produced at higher levels to protect proteins under vital circumstances in the multiple cellular mechanisms and stress such as hypoxia, oxidative damage, and hyperthermia [12]. Moreover, cancer cells also possess a favorable acidic microenvironment and hypoxic stability, which often helps eliminate detoxifying chemotherapeutic agents. Thus, the detoxifying chemotherapeutic agents result in recurrent tumors and metastases with increased malignancy and drug resistance [13,15]. 

The chemoresistance acquired by cancer cells has been classified into two types:

1. Intrinsic chemoresistance, which is acquired due to inherited characteristics of tumor cells such as genetic and phenotypic characteristics (genetic plasticity), molecular chaperones (heat shock proteins), and overexpression of anti-apoptotic proteins, detoxifying enzymes, and drug efflux transporters making them ideal to expel cytotoxic molecules.
2. Acquired chemoresistance, which is acquired due to prolonged treatment with a chemotherapeutic agent, results in the blockage of metabolic pathways associated with their pathogenesis, and miserably may frequently activate and enhance the alternative pathways, which leads to mutations in the tumor cells and tumor relapse for chemoresistance [16].

Furthermore, it becomes more complicated when cancer cells develop MDR to other structurally and mechanistically irrelevant drugs through a process called cross-resistance. MDR arises via overexpression of multidrug transporters or through altered apoptosis [17], which leads to lowered intracellular drug retention or modified tumor response. The combination of intrinsic and acquired resistance mechanisms mentioned above does not completely define chemotherapy drug resistance and indicates there are still unknown mechanisms contributing to cancer drug resistance that have yet to be discovered. Hence, there is a requirement for detailed investigation to understand the underlying mechanisms behind cancer drug resistance to provide the necessary information for designing future proper chemotherapy regimens to provide the treatment with an improved therapeutic index.

### 2.1. Inflammation and Drug Resistance

Inflammation is the non-specific immune system’s (innate immunity) response to any damage to cells caused by a physical, chemical, or biological factor. This includes infections caused by toxins and chemicals made by bacteria or viruses. These innate or nonspecific immune system responses, i.e., inflammation, help to commence the process of cellular repair and healing to treat the cellular damage produced [18]. The inflammatory response has been categorized into two types of the disease’s duration: acute and chronic inflammation. Acute inflammation is the initial response that happens before the immune response is formed and has a quick onset of minutes or hours [19]. Chronic inflammation, on the other hand, is a low-grade inflammatory response that endures from a few weeks to years because the cause of the inflammation persists. In general, the amount and severity of the injury relies on the origin of the damage as well as the body’s ability to recover and overcome the harm. Chronic inflammation is defined by the coordinated activation of many signaling pathways, the occurrence of acute phase reactants, and the rapid production of both aberrant cytokines and inflammatory mediators. [20]. This chronic inflammation often results in damage to healthy cells and tissues leading to multiple organ failure or even death in cancer and HIDs (hyperimmunoglobulin D syndrome) [21].

In 1863, the discovery of leucocytes in neoplastic tissues by Rudolf Ludwig Karl Virchow in his early work established the relation between inflammation and cancer. Hence, inflammation has been considered the primary etiological factor for tumorigenesis in some cancers [22,23,24]. For instance, the associations of human papillomavirus with cervical cancer, esophagus, and larynx cancers; asbestos-driven inflammation with bronchogenic carcinoma; the link between the infections caused by Helicobacter with the gastric adenocarcinoma; hepatitis B virus (HBV) infection associated with liver cirrhosis and hepatocellular carcinoma; and schistosoma-associated bladder carcinoma [25].

Various molecular pathways in the inflammatory process of cancer involve multiple cells and cytokines, which consist of innate immune cells including myeloid-derived suppressor cells (MDSCs), mast cells, neutrophils, tumor-associated macrophages (TAMs), natural killer (NK) cells, and dendritic cells (DCs), as well as adaptive immune cells including T-lymphocytes (T-cells) and B-lymphocytes (B-cells) [26]. These immune cells use multiple mechanisms to communicate them to regulate tumor growth, such as cytokine-mediated signaling and cell–cell direct contact. 

There are reports that consider the tumor’s microenvironment as a place where immune responses against tumors and inflammation caused by the tumor can coexist. Both of these factors must be in balance in order to figure out how the tumor will progress and develop [27]. There is growing evidence for demonstrating the most frequent existence of immune cells, such as T-cells and TAMs cells, within the tumor microenvironment (TME). TAMs cells contribute to angiogenesis, tumor growth, metastasis, and invasion [28]. Furthermore, T cells can exert both tumor-suppressive (T helper-1) and tumor-promoting effects (T regulatory), which are due to their ability to produce both immunosuppressive and proinflammatory cytokines. 

**Figure 1:**
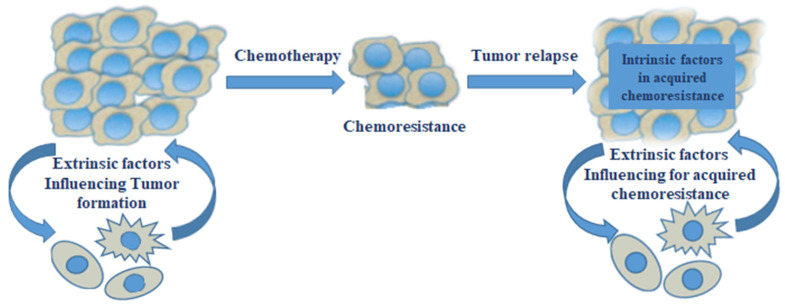
Cellular intrinsic mechanisms in cancer for acquiring drug resistance, such as the upregulation of inducible HSP expression, drug transporters, modified drug metabolism, and resistance to apoptosis/senescence pathways [29].

The progression of inflammation contributes to the distinct steps of tumor development, which include tumor promotion, initiation, malignant conversion, invasion, and metastasis. Inflammatory tumor microenvironment’s pivotal role in cancer development and progression is well documented. Significant progress has been made to better understand the mechanisms behind the immune system in modulating malignant outgrowth. There is increasing evidence not only for the role of the inflammatory tumor microenvironment in cancer development but also for the response of cancer to chemotherapy as well as acquiring chemoresistance, as shown in Figure 1 [29]. 

In addition, over recent years, there is growing evidence for the modulating role of the inflammatory tumor microenvironment and HSP expression of cancer cells in acquired drug resistance (Table 1).

The inflammation also affects the tumor drug response through different signaling pathways. The most prominent and widespread mechanism for drug resistance is the overexpression of membrane-anchored MDR transporters. However, the expression of these MDR transporters is regulated by inflammation and inflammatory mediators at numerous levels, including transcriptional, post-transcriptional, translational, and post-translational stages. For instance, P-glycoprotein (P-gp) is a transmembrane pump belonging to the ATP-binding cassette (ABC) family of transmembrane proteins whose expression has been found to be upregulated by cytokines [30]. The primary role of P-gp is likely to protect these susceptible organs from a diverse range of toxic compounds, including significant cancer chemotherapeutics, and its over-expression results in drug resistance [31]. Various signal pathways and transcription factors are familiar to impress the response to P-gp-mediated MDR, including the Jun kinase (JNK), extracellular signal-regulated kinase (ERK), PI3-kinase, p38-kinase, and protein kinase C signaling pathways; therefore, targeting these pathways may contribute new insight into the therapies for the treatment of ATP-binding cassette transporters B1 gene/P-gp-mediated (ABCB1/P-gp-mediated) MDR [32].

#### 2.1.1. Inflammatory Mediators and Multiple Drug Resistance: Cytokines

Cytokines are soluble proteins that influence and regulate a range of biological processes such as immune and inflammatory responses as signaling messengers [33,34]. Cytokines can be classified into a number of categories including colony-stimulating factors (CSF), chemokines, monokines, lymphokines, interleukins, and interferons based on their original cell sources and function. Cytokines can either promote or inhibit tumor development and progression, regardless of their cellular origin [35]. Inflammatory cytokines such as tumor necrosis factor-related apoptosis-inducing ligand (TRAIL), interferon-gamma (IFNγ), and interleukin-12 (IL-12) have been reported to demonstrate anti-tumor immunity. Epidermal growth factor receptor (EGFR), transforming growth factor-beta (TGF-β), and tumor necrosis factor-alpha (TNF-α) ligands were reported for stimulating cancer cell progression and survival. 

Whereas, TRAIL, perforin, granzyme B, and FasL have been reported to direct the signaling cytotoxicity of NK cells and cytotoxic T cells toward cancer cells. T helper cells have been reported to enhance the cytotoxic immunity effects by producing IFN-γ, and IL-17A. Dendritic cells (DCs), macrophages prime, NK, and T lymphocytes mediated response to tumor cells via the presentation of foreign antigens and the secretion of cytokines such as IL-12, IL-15, and IL-18. Alternatively, cytokines such as IL-6, IL-17, and IL-23 have been reported to enhance tumor progression. Further, cytokines have been reported to be stimulated by the activation of numerous downstream effectors, such as activator protein-1 (AP-1), nuclear factor-κB (NF-κB), caspases, signal transducer and activator of transcription (STAT), and Suppressor of Mothers Against Decapentaplegic (SMAD) transcription factors [36]. 

Tumor cells have been reported to produce chemokines and cytokines such as Regulated upon activation, normal T cell expressed and presumably secreted (RANTES), monocyte chemoattractant protein-1 (MCP-1), IL 6, IL 8, and IL 10, which have complex autocrine/paracrine signals. Pro-inflammatory cytokine IL-6 is reported to stimulate the generation of tumor cells and stimulate angiogenesis [37,38]. Some studies correlate the levels of IL-6 with progression and contrarily with the response to treatment and survival [37]. Possibly the most common mediator of chemoresistance mechanisms is IL-6, particularly, one of the most extensively dysregulated cytokines in cancer patients [39]. 

IL-8 is another pro-inflammatory cytokine belonging to the CXC chemokine family, which promotes tumor proliferation, vigorous angiogenic activity, and metastasis, stimulating the migration of monocytes, lymphocytes, and neutrophils [40,41]. IL-8 was overexpressed in ovarian cancer (OVCA) patients and the main role in the acquisition of chemoresistance reported against cisplatin and paclitaxel by increasing expression of both MDR1 and apoptosis inhibitory proteins such as Bcl-2, Bcl-xL, and XIAP and the activation of PI3 K/Akt, and Ras/MEK/ERK signaling [42]. Dabkeviciene et al. reported the role of IL-8 in acquired resistance to human colorectal carcinoma HCT116 cells [43]. IL-10 is a vital immunosuppressive cytokine that is generally overexpressed in tumors and protects cancer cells from immune-mediated destruction [44]. RANTES and MCP-1, which belong to the CC superfamily of chemokines, were found to promote tumor angiogenesis and might stimulate the migration of normal and malignant cells [45,46]. In MCF-7 cells, STAT3 phosphorylation was strongly induced by TRM-7 culture, and subsequent analysis exhibited increased levels of RANTES by cytokine antibody array assay. These experimental results validate the imbibition of STAT3 activation by adding neutralizing antibody, thereby decreasing the resistance of TRM7 cells to tamoxifen. Taken together STAT3-RANTES autocrine signaling is crucial for tamoxifen in human breast cancer cells [47]. Generally, this network of secreted signals tends to begin the progression of tumors and suppress cell function by enhancing the serious indication of cancer, such as perturbation of immune surveillance, chronic proliferation, chemoresistance, blood vessel recruitment, and prevention of apoptosis. The cytokines that promote or enhance the tumor microenvironment, which is accessible to tumor progression, and drug resistance have been tabulated in Table 2.

#### 2.1.2. Inflammatory Mediators and Multiple Drug Resistance: Cyclooxygenase

Prostaglandins (PGs) are a group of lipid mediators that play a pivotal role in regulating homeostatic functions and mediating pathogenic mechanisms, including inflammatory responses. These lipid-derived autacoids are created by the subsequent metabolism of arachidonic acid. The production of prostaglandins is found to be significantly elevated in organs that are inflamed by the activation of prostaglandin G/H synthases, colloquially known as cyclooxygenase (COX). Cyclooxygenase is a bi-functional enzyme having cyclooxygenase and peroxidase activity and occurs in two specific isoforms, mainly COX-1 and COX-2 [57]. COX-1 is the main source of prostanoids and is expressed constitutively in wide ranges of cells that subserve housekeeping functions, such as gastric epithelial cytoprotection and physiological homeostasis [58]. COX-2 isoenzyme is normally undetectable in most tissues (except kidney and CNS) but induced in response to inflammatory stimuli and mitogenic factors. COX-2, which is activated by inflammatory stimuli, cytokines, and tumor promoters, is an especially significant cause of prostanoid production during inflammatory as well as in proliferative diseases such as cancer [58]. Hence, COX-2 is considered to play a vital role during pathophysiological processes.

COX is a membrane-bound bifunctional enzyme having a vital role in converting arachidonic acid to prostaglandin G2 (PGG2) by the process of cyclooxygenation, followed by reducing the same, i.e., reduction of PGG2 to prostaglandin H2 (PGH2) by a typical peroxidase mechanism. PGH2 is the first intermediate substrate for the biosynthesis of all prostaglandins and for the thromboxanes. Prostacyclin (PGI2) is one of the most important cyclooxygenase products in the process of cyclooxygenation, and prostaglandin E2 (PGE2). Prostaglandins activate cancer cell production, enhance angiogenesis, suppress apoptosis, and strengthen metastatic potential. The expression of COX-2 was reported to be upregulated in various cancers such as gastric, colon, pancreatic, prostate, breast, esophageal, bladder, lung, and neck cancers [59].

The overexpression of COX-2 has been reported due to multiple stimuli, including in response to oncogenes (like HER-2/neu); tumor simulator molecules such as ceramides, vasoactive peptides, bile acids, phorbol esters, endothelin-1, and methanandamide; growth factors (like EGF); pro-inflammatory cytokines such as TNF-α, IL-1β, NF-IL6, NF-κB, and AP-1; bacterial lipopolysaccharide; nuclear factor of activated T cells (NFAT); and viral infection (polyomavirus enhancer activator-3 (PEA3)). The overexpression of COX-2 is necessary when the stability of both transcription activation and mRNA occurs simultaneously. However, the determination of COX-2 levels in neoplastic tissues is explicitly distressed by post-transcriptional regulation as the presence of AU-rich elements (ARE) in the 3ˈ-UTR region of COX-2 transcripts greatly influences the strength of mRNA. The mRNA stability and COX-2 transcripts are upregulated by cytokines, oncogenes, tumor promoters, growth factors, ERK1, ERK 2, and p38. The rate of protein synthesis and degradation was also found to greatly influence the expression of COX-2 [60]. 

Although multiple factors for enhancing the transcription of COX-2 were reported, there are also reports on the negative modulators for COX-2 expression. The association of the 3ˈ-UTR region of mRNA of COX-2 with some elements reported inhibiting its transcriptional. In cancers, the hypermethylation of specific promoter regions of the COX-2 gene is reported to down-regulate its expression [61]. Similarly, the bioactivity, as well as the expression of COX-2, was inhibited by multiple anti-inflammatory cytokines (for instance, IL-4, IL-10, and TGF-b) and steroidal and non-steroidal agents. Like many inflammatory mediators, COX-2 also demonstrated precise effects on the expression of MDR. There are increasing reports about the elevated expression of COX-2 related to the level of MDR proteins (e.g., MDR1, BCRP, MRP1, MRP2, and MRP3), and they actively interfere in the chemotherapy outcome in cancer patients [62]. For instance, MDR-1b gene expression is significantly induced by prostaglandin E2 (PGE2) and prostaglandin-F2a (PGF2a) [63]. The overexpression of COX-2 in TR-5 colon cancer cells has been found to induce the MRP1 expression and result in chemoresistance to cisplatin [63]. However, the chemosensitivity of TR-5 cells to cisplatin resorted by pretreating with the COX-2 selective inhibitor JTE-522 [63]. Further, the bioactivity and expression of BCRP have increased due to TPA-induced expression of COX-2 in distinct breast cancer cells. However, several studies also exhibit the differences between COX-2 level and MDR expression. For instance, in human NCI-H460 large-cell lung cancer, the enforced expression of COX-2 was found to not affect the activity and expression of MRP1 and P-gp. At the same time, the negative correlation between COX-2 and MDR expression has also been in medullary thyroid carcinoma cells [64,65]. However, there are substantial differences between inflammatory responses and MDR regulations among different types of cells.

### 2.2. Heat Shock Protein and Immune System

HSPs play a vital role in the functioning of the immune system [66,67], in addition to their functioning as molecular chaperones where responsible for protein folding, assembly, stability, translocation, and degradation. Nevertheless, the relationship between HSPs and immune cells is complex, and the specific functions of each component within the immune system might vary based on the particular type of HSP and immune cell being examined.

#### 2.2.1. HSPs and Their Various Roles in Immune System

The outlines of the divergent roles of HSPs throughout the immune system are as follows:

**Antigen Presentation:** The process of antigen presentation is a crucial component of the immune response. HSPs are essential in the mechanism of antigen presentation to immune cells, therefore indicating their involvement in the immunological response. Significantly, HSP70 and HSP90 proteins function as molecular chaperones, facilitating the proper folding and transport of antigens within cellular environments. Antigen-presenting cells (APCs), such as dendritic cells, possess the ability to recognize antigens when they are presented on the cell surface in this manner. This stage holds significant importance during the initiation of immune responses.
**Immune Cell Activation:** The activation of immune cells, including dendritic cells, macrophages, and T cells, can be facilitated by HSPs within the human body. When released by cells under stress or early apoptotic stage, HSP possesses the capacity to act as danger signals, commonly referred to as damage-associated molecular patterns (DAMPs). This phenomenon possesses the capacity to elicit immunological responses, hence aiding in the elimination of cancer cells or other detrimental microorganisms.
**Antigen Processing:** HSPs are found in higher amounts in some cancer cells due to the cellular stress associated with tumor growth. Tumor-derived HSPs can serve as antigens that activate immune responses against cancer cells. In this context, HSP-based vaccines have been explored as potential cancer immunotherapies.
**Tumor Immunity:** The presence of cellular stress associated with tumor development leads to an increased abundance of HSPs in certain cancer cells. This factor is a contributing element to tumor immunity. Tumor-derived HSPs possess the capacity to function as antigens, thereby eliciting immune responses specifically targeting malignant cells. In the context of this approach, there has been research conducted on HSP-based vaccinations with the aim of exploring their potential use in cancer therapy.

#### 2.2.2. The Non-Specific Targeting of HSPs and Its Potential Side Effects

The non-specific targeting of heat shock proteins can elicit unforeseen consequences on the immune system in the following ways [68].

**Immunosuppression:** Immunosuppression refers to the phenomenon wherein tumors exhibit an excessive production of HSPs, which can potentially aid in evading immune surveillance. There is a potential for enhancing immune responses against malignancies by altering this system by non-specific targeting of HSPs. Nevertheless, it is plausible for it to exert an impact on healthy cells, hence inducing immunosuppressive effects.
**Autoimmune Reactions:** The non-selective targeting of HSPs may lead to the occurrence of autoimmune responses, as HSPs are found in both good and unhealthy cells [69]. The phenomenon may lead to undesirable immune reactions that target and damage healthy tissue.
**Differential Expression:** Various HSPs have diverse roles throughout the immune response. Nevertheless, the expression of these HSPs may vary depending on the specific cellular context in which they are located. Non-specific targeting has the capacity to disrupt the balance among highly sensitive persons, perhaps leading to unanticipated consequences.
**Complexity of Tumor Microenvironment:** The microenvironment associated with tumors exhibits a notable level of intricacy, characterized by its substantial heterogeneity and the presence of several immune cell populations engaged in complicated intercellular interactions [70]. The lack of specificity in targeting HSPs carries the potential drawback of inadequate efficacy in combating the many immune evasion mechanisms employed by different types of cancer.

### 2.3. Heat Shock Protein and Multiple Drug Resistance

In cancer cells, an elevated level of protein synthesis is required because of their rapid proliferation, leading to highly rewired metabolic stress and signal transduction pathways. Hence, cancer cells become more dependent on a class of proteins called stress-inducible heat shock protein (HSP) whose primary activity is as a molecular chaperone, which means that they mediate protein folding to the proper native form. HSP are ubiquitous polypeptide-proteins in all prokaryotic and eukaryotic cells [71]. HSP is the essential cellular machinery to maintain cellular homeostasis under standard and detrimental and adverse growth conditions, such as in cancer cells [72,73]. Thus, the cytoprotective functions of HSP become necessary to maintain the homeostasis of the cancer cell for its survival. 

According to molecular function and mass, HSPs are categorized into six families according to their weight: chaperonin (HSPD/E and CCT); HSPB; HSP40 or DNAJ; HSP70 or HSPA; HSP90 or HSPC; and HSP110 or HSPH. However, the expression and activity of HSP27, HSP60, HSP70, or HSP90 were found to be exceptionally high in cancer cells, suggesting the extremely vital role of these HSPs in the regulation of cellular responses and functions during carcinogenesis [74].

Furthermore, the level of HSP expression in cancer cells has further increased due to exposure to various death stimuli, including chemotherapeutic drugs, oxidative stress, hyperthermia, radiation, and soon [75]. These indicate the correlation of cancer cells’ resistance to chemotherapy with the enhanced expression of HSPs. For example, HSP27, an inductive HSP family, has been demonstrated to generate pharmacological resistance to dexamethasone in cells of multiple myeloma by restricting the release of a second mitochondria-derived caspase activator (SMAC), which is an additional crucial parameter for apoptotic regulation [76]. The overexpression and accumulation of HSP27 are associated with multiple characteristics of carcinogenesis, including elevated cytoprotection, inhibition of apoptotic cell death, and multidrug resistance, through direct interaction of apoptotic proteins, leading to a poor prognosis of various cancers [77]. 

Similarly, HSP70s play an essential role in tumor progression that is generally found at elevated levels in cancer cells, especially in their stress-inducible isoform of HSP70, such as HSP70-1-A1, HSP70-1, or HSP72. HSP70 overexpression is related not only to promoting cancer development and metastasis, but, more importantly, to drug resistance and poor patient prognosis in cancer [78]. Understanding the underlying molecular mechanisms of HSP70 in cancer development reveals its pivotal role in inhibiting apoptosis in both intrinsic and extrinsic pathways at multiple points. In the intrinsic pathway, Bax activation is inhibited by HSP70 and blocks the permeabilization of the mitochondrial membrane, thereby releasing pro-apoptotic factors [79]. At the same time, HSP70 stops the death-inducing signaling complex (DISC) in the extrinsic pathway [80]. The significant role of HSP60 has also been found to contribute to cancer progression and acquired drug resistance of cancer cells. Therefore, HSP60 has been used as a therapeutic target in cancer treatment; for example, reversal of 5-fluorouracil drug resistance was observed in SW480 colorectal cancer cells when the expression of HSP60 inhibited in 5-fluorouracil-resistant SW480 colorectal cancer cells showed after [81]. 

Thus, the expression of various HSPs like HSP27 has a crucial role in a cancer cell acquiring drug resistance. Therefore, HSP is an ideal therapeutic target for cancer treatment to overcome drug resistance [82]. For example, RP101 is an antiviral nucleoside, and HSP27 inhibitors are reported to function as a chemo-sensitizing agent in preventing the progression of drug resistance against multiple anticancer drugs. The binding of RP101 Phe29 and Phe33 of HSP27 through Phe29 and Phe33 of HSP27 was reported to affect the ant apoptosis properties of HSP27 by weakening the binding of HSP27 to cytochrome C, pro-caspase3, and Akt1 [83]. In stage III and IV clinical studies of patients with pancreatic cancer, the overall survival rate increased by 8.5 months when treated with RP101 compared to the historical control group (NCT00550004) [83].

## 3. Heat Shock Proteins and their Immunomodulatory Effect in the Tumor Microenvironment, Drug Resistance, and Treatment

HSP overexpression contributes to tumor development and progression and in determining their response to various treatments, including chemotherapy. For example, as discussed above, HSP27 overexpression has contributed to the poor prognosis of osteosarcomas and gastric carcinomas [84].

**Figure 2:**
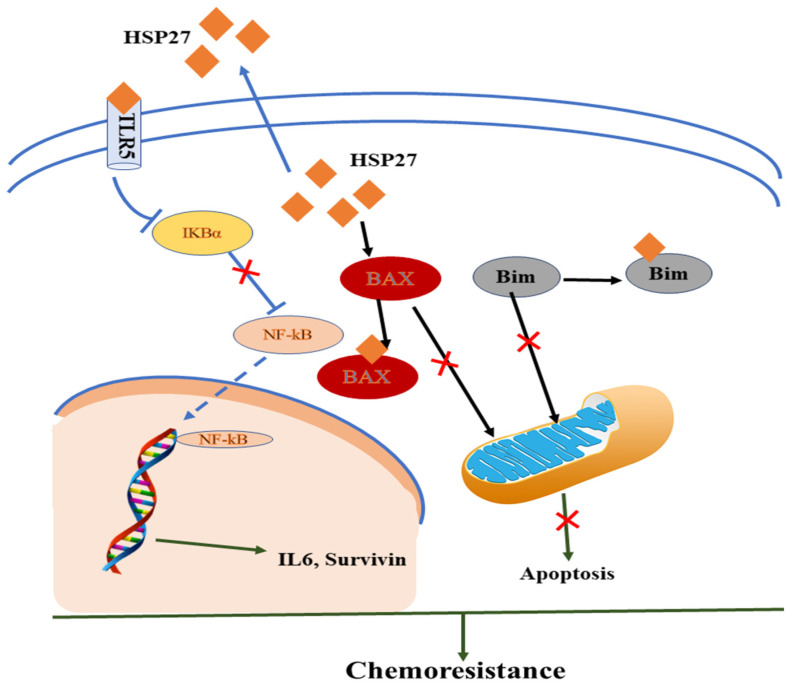
Mechanistic representation of HSP and immunomodulatory effect in the tumor microenvironment in acquiring chemoresistance.

Zheng et al. (2018) recently showed that HSP27 played an important role in SCCT cells for chemoresistance via synergistic extracellular and intracellular signaling [85]. In the intrinsic pathway, the reduction of TLR5 or the restored IκBα protein level of IB proteins interrupts the transactivation of extracellular HSP27-induced NF-κB transactivation resulting in drug resistance. In the extrinsic pathway, intracellular HSP27 interacts with BAX and BIM to suppress their translocation to the mitochondrion, thus blocking the successive release of cytochrome C and preventing cells from apoptosis by communicating with Cyto C [85,86,87]. HSP27 exhibited biological effects on monocytes, producing IL10 and TNFα and blocking monocyte differentiation to normal dendritic cells and tumor-associated macrophages to stimulate cancer progression and chemoresistance as shown in Figure 2 [85,88].

However, there are also reports stating the increase in HSP expression in improving chemotherapy outcome, such as in the case of HSP70 in osteosarcomas, where enhanced expression of HSP70 was found to significantly influence the prognosis of breast cancer [89]. Furthermore, the circulating levels of HSPs along with their antibodies in blood provide attractive biomarkers for analyzing stage and aggressiveness in various types of cancer and monitoring the treatment process during treatment due to the extraordinary relationship between HSPs and drug resistance. More importantly, one of the immune system’s essential functions in tumor prevention is tumor immune surveillance, which particularly recognizes and eradicates cancerous and/or precancerous tumors by analyzing the expression level of tumor-specific antigens or molecules that are induced as a result of cellular stress. In the tumor microenvironment, some HSP plays a critical role in managing the delicate balance of protective and destructive immunological responses; thus, it is quite evident that HSP possesses a central understanding role in oncoimmunology. Thus, the expression or activity of HSP modulation provides a new therapeutic strategy in cancer immunotherapy [90]. 

The application of HSP-based immunotherapeutic approaches may contribute enormously to the field of oncoimmunology to obtain effective anticancer regimens. For example, antitumor T-cell immunity might be enhanced by the application of tumor-based HSP90 inhibition. HSP90 has essential functional roles that complement the role in innate and adaptive immune responses, including activation/maturation of dendritic cell antigen presentation, lymphocyte activation, and cross-priming [91,92]. Thus, targeted inhibition of HSP90 can stimulate a dual regulatory role in both immunosuppressive and immunostimulatory effects. Accumulating evidence suggests that T-cell-mediated antitumor immunity promotes increased tumor immune surveillance and recognition through therapeutic exploitation of client proteins’ dependent and independent mechanisms. 

The enhancement in the anti-tumor T-cell immunity using tumor-based HSP90 inhibition has been reported. In addition, the invention that oncoprotein-directed dysregulation of PD-1/PD-L1 signaling can occur as an adaptive response for endogenous antitumor immunity elevates the capability of upstream interference via HSP90 blockade. However, the mechanism to integrate the process of HSP90 inhibition and blockade of immune checkpoints should be investigated to improve antitumor immune responses. Expect the above therapeutic approach, i.e., immune-checkpoint inhibitors for cancer immuno-therapy, and many other approaches such as cytokines, immune-checkpoint inhibitors, targeted antibody therapies, and adoptive cell transfer methodologies. Overall, compelling evidence suggests that HSPs are attractive targets for cancer immunotherapy.

## 4. Other HSP Immunostimulatory Properties-based Cancer Immunotherapy

The following unique immunostimulatory properties of HSP that can be used as physiological adjuvants for cancer immunotherapy are as follows [19]:

- Assuring the desired specificity and sensitivity of antigen targeting possibly assured through receptor-mediated uptake of molecular chaperones via antigen-presenting cells (APCs).
- Molecular chaperones are able to serve as danger signals and stimulate innate immune components for the progression of active immune responses.

These novel immunostimulatory properties of HSPs facilitate their utilization as adjuvants to develop various cancer immunotherapy approaches.

### 4.1. Tumor-based HSP Vaccine

The HSP possesses substantial properties that can be utilized for targeting dendritic cells (DCs) and can also used as a natural adjuvant for inducing adaptive immune responses based on the ability of HSP to deliver multiple antigens and serve as the secure constituent of current vaccines [93,94]. Under stress conditions or immunological danger signals, HSP-bound tumoral peptides could be released in the extracellular medium owing to the chaperone activity of HSP [19]. The interaction between HSP and APC cells involves multiple receptors, including CD40, CD91, and LOX-1. After endocytosis of HSP–peptide complexes, they were humiliated and subsequently led to cross-presentation of the tumor peptide to CD8+ T cells through the major histocompatibility complex 1 (MHC-1) molecules [95]. Vitespen is an autologous tumor-derived HSP Gp96, which has shown an excellent clinical impact for treatments of kidney cancer and melanoma in phase III clinical trials [96]. Generally, the immunogenicity of HSPs is primarily because of its two essential properties: first, their peptide-dependent capacity to stimulate and elicit adaptive CTL responses against antigenic peptides and, second, their peptide-independent immunomodulatory capacity.

Several reports have shown that particular HSPs, including HSP70 and Gp96, are effective carrier molecules for cross-presenting [97]. The few HSP-based vaccines that are under clinical trials for tumor treatment have been summarized in Table 3.

### 4.2. HSP as an Antigen for Immune Responses Stimulation

The immune system can be stimulated for cancer treatment using HSP as an antigen without chaperonin activity [19]. For example, intratumoral vaccination with a recombinant oncolytic adenovirus overexpressing HSP, in particular HSP70, thus reducing tumor growth [104]. Li et al. (2014) reported that HSP peptide-specific CTL (cytotoxic T lymphocytes) effectively reduced tumor burden in the mouse model of a myeloma xenograft. Treatment of CTLs engineered to target HSP27 and HSP90 peptides effectively decreased tumor growth in a mouse model with myeloma xenograft and stimulation of peripheral blood mononuclear cells (PBMC), resulting specifically in the generation of HSP peptide CTL [105].

### 4.3. HSP as an Adjuvant-Free Carrier to Stimulate Immune Responses

Adjuvants are substances that can enhance, stimulate, and modulate innate immune responses, as well as alter the quality and quantity of adaptive immune responses [94]. HSP was used as an adjuvant for stimulating the immune response of CTLs in various cancers and infectious diseases. Some reports revealed that immune activities reside within N- or C-terminal fragments. Thus, a small piece or part of HSP as an adjuvant in vaccines for cancer prevention can be used [106]. For instance, the N-terminal piece of Gp96 (NT-gp96) has been reported as a potential adjuvant that has been found to increase specific immune responses of CTLs against hepatitis B virus (HBV) and hepatocellular carcinoma infections [106,107]. 

The anti-tumor responses based on targeting the HSP70 family of fusion proteins and tumor-associated antigens (TAAs) were evaluated by Zhang and Huang, who reported that the C-terminal domain of HSP70 seemed to be the essential part of eliciting anti-tumor responses, including NK cell stimulation against B16 murine melanoma expressing tumor-associated antigens [108]. The investigation further proves the importance of the peptide-binding domain that mediates the binding of HSP70 and generated DNA encoding the E7/HSP70 vaccine. However, in conjunction with the HSP70 functional domain, the orientation of links between HSP70 and HPVE7 also has significant clinical importance in optimizing HSP70-based DNA vaccines [109]. 

In chronic non-progressive pneumonia, membrane-associated proteins elongation factor Tu and Mycoplasma ovipneumoniae elongation factor HSP70 induce significant levels of cytokines, such as TNF-α, IFN-γ, IgG, IL-4, IL-5, IL-6, and IL-12 [19]. In fact, recombinant HSP70 has the potential to act as a Th1 cytokine-like adjuvant in mice [110]. A similar study has been conducted using Small HSP, that is, HSP27 as an efficient adjuvant to improve HIV-1Nef antigen-specific immunity. A fusion protein, namely HSP27-Nef, secured from the fusion of HIV-1Nef and HSP27, significantly improved the Nef-specific T-cell response. In fact, these regimens induced high levels of IgG2a and IFN-γ, which are Th1-associated cytokines, as well as Granzyme B secretion. Thus, this study demonstrated the immunostimulatory properties of HSP27, which can be utilized for diverse immunization approaches other than Freund’s adjuvant, and these findings suggested that HSP27 can be used as an effective adjuvant in protein-based vaccines to boost HIV-1 Nef-specific B- and T-cell immune responses [111] and serve as a promising HIV-1 vaccine candidate also [112].

## 5. Conclusions

Despite the advancement in the field of cancer therapeutics, multidrug resistance has become one of the significant hurdles causing a detrimental effect on the chemotherapy regimens leading to poor prognosis and recurrence of cancer. Considerable efforts have been made to understand the mechanisms behind the chemoresistance. There is growing evidence to support the tight relationship between inflammation and HSP for the incidence of cancer invasion and metastasis, along with the development of multidrug resistance. For instance, the infection might trigger a complex cytological process, including abnormal cell proliferation and subsequent inflammation to provide the adequate tumor microenvironment for cancer development and drug resistance involving several heat-shocks in these complex scenarios. Unraveling the molecular mechanisms behind mediating the immunomodulatory effect of HSP as immunostimulatory (induction) and immunosuppressive (regulatory activity) provides a new insight clinically for developing better strategies in the relevance of HSPs as therapeutic targets or agents for fighting drug resistance in cancer with improved efficacy.

## Figures and Tables

**Table 1:** The role of HSP and inflammatory tumor microenvironment supporting tumor progression and acquiring drug resistance.

Inflammatory Tumor Microenvironment and HSP Supporting Tumor Formation and Progression	The Role of HSP and Inflammatory Tumor Microenvironment Supporting the Acquiring Drug Resistance
Tumor microenvironment remodeling by recruitment of leukocytes, lymphocytes, expression of tumor-promoting chemokines, cytokines, and induction of an angiogenic switch.	Overexpressed pro-inflammatory cytokine-like IL-8 helps in the acquisition of the chemoresistance reported against cisplatin and paclitaxel by increasing expression of both MDR1 and apoptosis inhibitory proteins (Bcl-2, Bcl-xL, and XIAP), and the activation of PI3 K/Akt and Ras/MEK/ERK signaling.
Activated inflammatory cells serve as sources of reactive nitrogen intermediates (RNI) and reactive oxygen species (ROS) for inducing DNA damage and genomic instability essential for cancer progression.	Difference in pharmacokinetics and pharmacodynamics of drugs in vitro and in vivo.
Tumor-promoting cytokines production like TNF-α activates the transcription factors, in pre-malignant cells such as NF-κB, STAT3, and AP-1, and induces genes to stimulate cell proliferation and survival.	The expression of stress-inducible HSP inhibition of apoptosis in both the intrinsic and extrinsic pathways at multiple points like HSP70 inhibits Bax activation and blocks the permeabilization mitochondrial membrane in the intrinsic pathway, thereby proapoptotic factors release. Whereas HSP70 stops the death-inducing signaling complex assembly (DISC) in the extrinsic pathway
Extensive stress-inducible HSP production in cancer for regulating highly rewired metabolic stress	

**Table 2:** Various cytokines and the mechanism involved in acquiring the chemoresistance is tabulated as follows.

Cytokine	Cancer Type	Drug	Mechanism	Ref.
IL-6	Prostate Cancer	Enzalutamide	IL-6 expression helps the constitutive activation of Stat3 leading to blocks of enzalutamide-mediated apoptosis	[48]
IL-17	Colorectal Carcinoma	Cisplatin	IL-17 expression apoptosis-related proteins, including serine/threonine-protein kinase mTOR, apoptosis regulator BAX, phosphorylated apoptosis regulator Bcl-2, and protein kinase B (p-Akt), thereby inhibiting cancer cell apoptosis through targeting mTOR, Bax, Bcl-2, and p-Akt.	[49]
IL-4 and Il-10	Thyroid cancer	Cisplatinum, Doxorubicin, Taxol	Autocrine production of IL-4 and IL-10 promotes tumor progression and helps in arriving at drug resistance via the up-regulation of antiapoptotic proteins like Bcl-2 and Bcl-x.	[50]
IL-6 and IL-8	Breast cancer	Paclitaxel, Doxorubicin	Associated with the expression of P-gp a transmembrane pump required for MDR phenotype by drug efflux	[51]
IL-8	Breast cancer	Paclitaxel	The paracrine effect of IL-8 release helped to acquire TLR4-mediated paclitaxel resistance	[52]
IL-8	Skin cancer	Trametinib	Molecular and cellular changes in trametinib-resistant cell lines	[53]
Transmembrane tumour necrosis factor-α (tmTNF-α)	Breast cancer	Doxorubicin	tmTNF-α found to activate the ERK/GST-π axis and NF-κB pathway through reverse signaling to mediate DOX resistance	[54]
Epidermal growth factor receptor (EGFR)	Breast cancer		EGFR promoted the activation of the Raf/MEK/ERK and PI3K/AKT signal transduction pathways responsible for drug resistance.	[55]
Autocrine motility factor (AMF)	Fibrosarcoma	Mitomycin C	AMF administration inhibited expression of the apoptotic protease activating factor-1(APAF-1) and Caspase-9 genes encoding the proteins required for the formation of “apoptosome” complex	[56]

**Table 3:** Various vaccine-based HSPs currently under clinical trials for cancer immunotherapy [19].

Vaccine-Based HSPs	Targeted Cancer	Present Clinical Trials	Ref.
Vitepsin: Gp96-based vaccine	liver, ovarian, glioma, melanoma	Phases II and III clinical trials have been approved	[98]
HPV16oE7-HSP70-based vaccine	Cervical cancer	The preclinical study is underway	[99]
HSP. 70PC-HSP70-based vaccine	Breast cancer	Under phase I clinical study	[100]
HSP.PC-96-Gp96-based vaccine	Renal carcinoma	Under phase II and phase III clinical trials	[101]
HSP70 activated NK cells	Colon cancer and NSCLC	Under phase I and phase II clinical status	[102,103]
